# Sequential evaluation of *CALR* mutant burden in patients with myeloproliferative neoplasms

**DOI:** 10.18632/oncotarget.16797

**Published:** 2017-04-03

**Authors:** Chiara Cavalloni, Elisa Rumi, Virginia V. Ferretti, Daniela Pietra, Elisa Roncoroni, Marta Bellini, Michele Ciboddo, Ilaria C. Casetti, Benedetta Landini, Elena Fugazza, Daniela Troletti, Cesare Astori, Mario Cazzola

**Affiliations:** ^1^ Department of Molecular Medicine, University of Pavia, Pavia, Italy; ^2^ Department of Hematology Oncology, Fondazione Istituto di Ricovero e Cura a Carattere Scientifico (IRCCS) Policlinico San Matteo, Pavia, Italy

**Keywords:** myeloproliferative, burden, CALR, JAK2, sequential

## Abstract

We investigated the variation of *CALR*-mutant burden during follow-up in 105 *CALR*-mutant MPN and compared it to the variation of *JAK2*-mutant burden in 226 *JAK2*-mutant MPN.

The median allele burden at last evaluation was significantly higher than at first evaluation in essential thrombocythemia (ET) (49.5% vs 45%, *P* < .001) but not in primary myelofibrosis (PMF) (52% vs 51%, *P* 0.398). Median values of slope were positive both in ET (0.071) and in PMF (0.032). In *CALR*-mutant ET there was a difference between natural and therapy-related slope (*P* 0.006).

In the *JAK2*-mutated cohort, the median allele burden at last evaluation was not different respect to that at first evaluation, neither in ET (22.9% vs 23.2%, *P* = 0.216) nor in PMF (50.5% vs 45.0%, *P* = 0.809), despite a positive slope. Multivariate analysis to evaluate the effect of mutation (*CALR* vs *JAK2*) on the slope of mutant burden in not treated pts with a positive slope adjusting for diagnosis (ET vs PMF) showed a trend toward a higher increase of mutant burden in *CALR* vs *JAK2* (β = 0.19, *P* = 0.061) with no difference between diagnosis (*P* = 0.419). The findings of this study suggest that clonal expansion in CALR-mutant MPN is faster than that observed in *JAK2*-mutant MPN.

## INTRODUCTION

The three classic Philadelphia-negative myeloproliferative neoplasms (MPN), polycythemia vera (PV), essential thrombocythemia (ET) and primary myelofibrosis (PMF), are clonal hematopoietic disorders that cause expansion of one or more myeloid lineages [1].

Most patients with ET or PMF not associated with *JAK2* or *MPL* alterations carry somatic mutations of *CALR* [2, 3]. The clinical course of *CALR*-mutated patients is more indolent than that of *JAK2*-mutated patients, having a lower risk of thrombosis in ET and a better survival in PMF [4–7]. Previous studies have reported that most *CALR* mutations are heterozygous [2, 3].

Data regarding sequential evaluations of *CALR*-mutant burden during follow-up are still lacking. In this study we aimed to investigate the variation of *CALR*-mutant burden during follow-up in *CALR*-mutant MPN and to compare it to the variation of *JAK2* V617F-mutant burden in *JAK2*-mutant MPN matched for diagnosis.

## RESULTS AND DISCUSSION

All 105 *CALR*-mutated patients remained positive at second evaluation: among the five secondary acute myeloid leukemia (AML) evolved from *CALR*-mutant MPN no cases of *CALR* negative AML was observed. This is in contrast with the observation that *JAK2*-mutant MPN frequently yields *JAK2* negative AML [8, 9].

**Figure 1:**
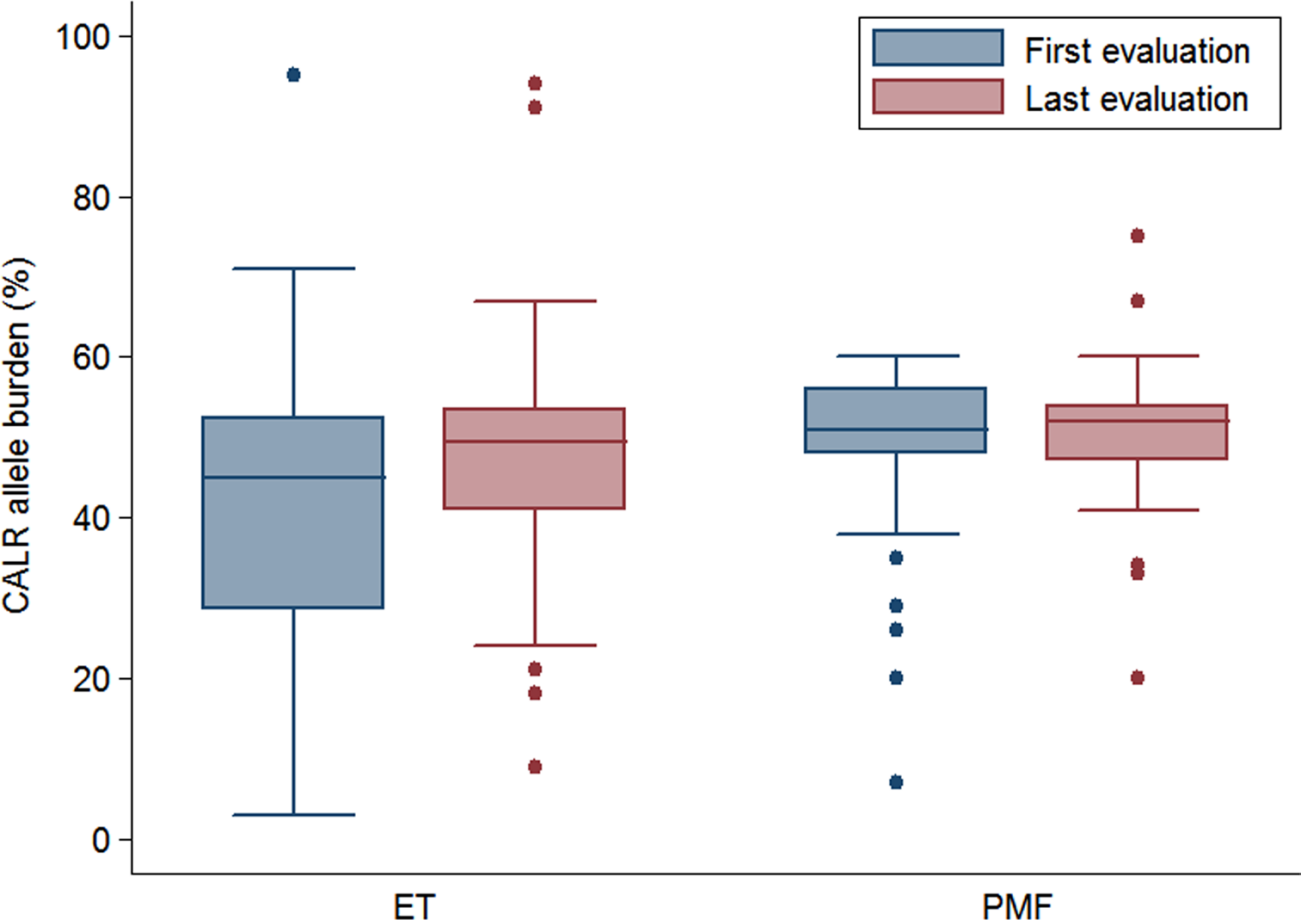
*CALR* allele burden *CALR* allele burden (%) at first and last evaluation in patients with essential thrombocythemia (ET) and primary myelofibrosis (PMF).

In *CALR*-mutant ET the median allele burden at last evaluation was higher than at first evaluation (49.5%, range 9–94% vs 45%, range 3%–95%, *P* < .001). In *CALR*-mutant PMF the median allele burden at last evaluation (52%, range 20–75%) did not significantly differ from the median allele burden at first evaluation (51%, range 7–60%, *P* 0.398). *CALR* allele burden at first and last evaluation in ET and PMF are reported in Figure 1.

Median values of slope were positive both in ET (0.071 increase of *CALR* mutant alleles/month, range −1.114;2.789) and in PMF (0.032 increase of *CALR* mutant alleles/month, range −5.526;1.229), without a significant difference between the two diagnosis (*P* 0.319). Taken together these data suggest a progressive increase of the clone during time.

Type 1-like and type 2-like *CALR* mutations did not differ in terms of baseline values and evolutionary pattern. The baseline values did not differ both considering only the subgroup of patients assessed at clinical diagnosis (35% in type 1-like vs 38.5% in type 2-like, *P* 0.863) and considering all patients (48.5% in type 1-like vs 46.5% in type 2-like, *P* 0.463). Also the slope was not different (0.060 in type 1-like, 0.059 in type 2-like, *P* 0.340).

**Figure 2:**
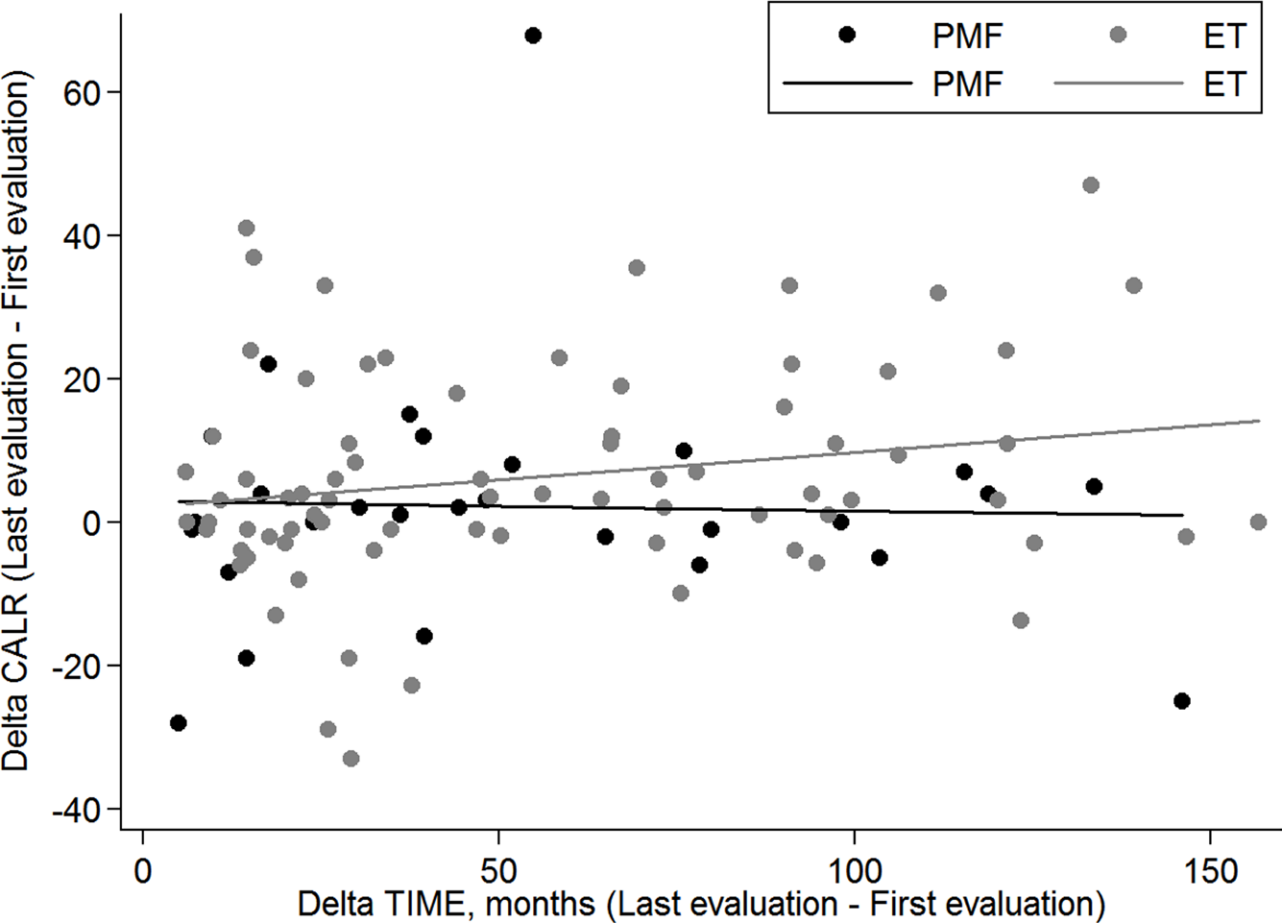
Scatter plot of Delta *CALR* according to Delta time The Delta *CALR* (difference between last and first *CALR* allele burden evaluation) according to Delta time (time between last and first evaluation) in ET (grey dots) and PMF (black dots). In PMF patients the Delta *CALR* does not significantly change with the increasing of interval time between first and last evaluation (beta = −0.01 *P* = .866); in ET patients the Delta *CALR* increases (with borderline significance) with the increasing of interval time between first and last evaluation (beta = 0.08 *P* = .074).

The delta *CALR* (difference between last and first *CALR* allele burden evaluation) according to time interval between first and last evaluation in ET and PMF is reported in Figure 2.

**Figure 3:**
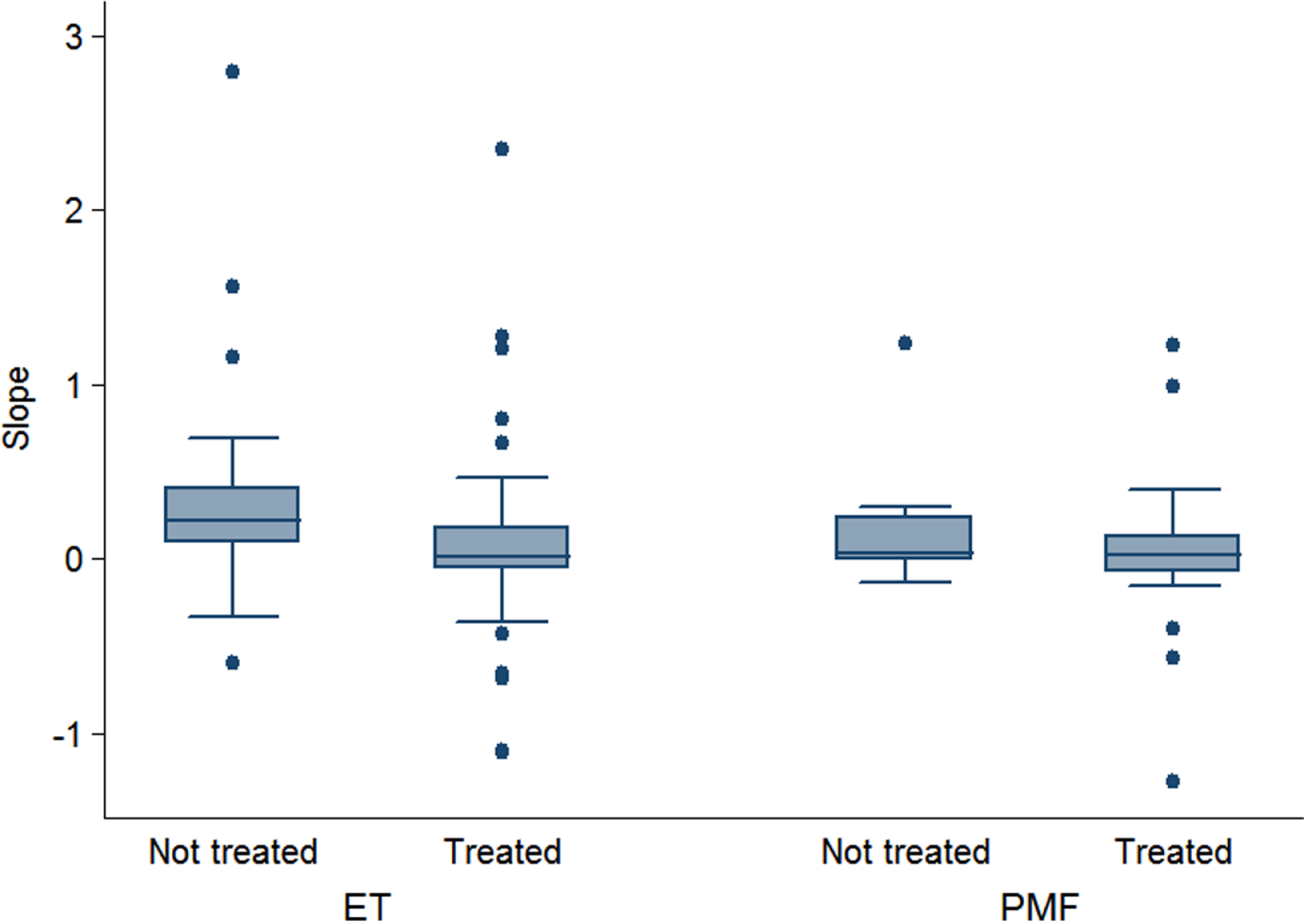
Slope of the *CALR* allele burden Slope of *CALR* mutant alleles between sequential evaluations in patients with essential thrombocythemia (ET) and primary myelofibrosis (PMF). The left box-plot shows the natural slope obtained in patients who did not receive cytoreduction; the right box-plot shows the therapy-related slope obtained in patients who receive a myelosuppression. Mann Whitney *U* test showed a difference between natural and therapy-related slope in ET (*P* 0.006) but not in PMF (*P* 0.451) One treated PMF outlier patient (−5.526) was not represented for graphic reasons.

To investigate whether cytoreductive treatment might influence the mutant allele burden we divided patients in “Treated” (72 patients) if they had received at least one cytoreductive treatment before or between the two sequential evaluations and “Untreated” (33 patients) if they did not receive any treatment. Mann Whitney *U* test showed a difference between natural (0.178) and therapy-related slope (0.015) (*P* 0.005) in *CALR*-mutated patients. When repeating the same analysis in the two disorders separately, we confirmed a difference between natural (0.207, range −0.595;2.789) and therapy-related slope (0.015, range −1.114;2.352) in ET (*P* 0.006) but not in PMF (0.033, range −0.138;1.229 vs 0.012, range −5.526;1.22, *P* 0.451). The slope of *CALR* allele burden in treated and untreated ET and PMF is reported in Figure 3. To evaluate whether the difference between natural and therapy-related slope observed in ET might be due to the effect of interferon on the allele burden we compared the slope of ET patients treated with interferon (*N* = 7) with the slope of ET patients treated with other cytoreductive drugs (*N* = 45) but we did not find a statistically significant difference (*P* = .0883). Two *CALR*-mutated patients underwent allogeneic bone marrow transplantation (BMT) and obtained complete molecular remission at the first evaluation performed 100 days after BMT.

Then we evaluated whether the increase of the allele burden in ET patients might depend upon disease evolution. We compared the slope of ET patients with disease evolution, including 6 patients who developed post-ET myelofibrosis and 3 patients who progressed to acute myeloid leukemia, with the slope of ET patients without disease evolution. We did not observe a difference between ET patients who developed and those who did not develop disease progression, both in patients with positive slope (*P* 0.329) and in patients with negative slope (*P* 0.280). The expansion of the *CALR*-mutated clone during time seems to occur independently from disease progression.

It has been previously shown that patients with high *CALR*-mutant burden had acquired copy neutral loss of heterozygosity of chromosome 19p, involving transition from heterozygosity to homozygosity for the *CALR* mutation [2]. At any rate 19pLOH appears to be relatively uncommon event [4], in contrast with 9pLOH in *JAK2*-mutant MPN and 1pLOH in *MPL*-mutant MPN [10, 11]. Accordingly, in our study cohort, only five *CALR*-mutated patients had a mutant allele burden equal or greater than 75%. In addition, the genotyping of the rs1049481 SNP in these cases was not informative in four patients, because of homozygosity both in granulocytes and in T lymphocytes. The single informative patient (heterozygous in T lymphocytes) showed homozygous granulocytes, thus confirming 19pLOH, likely as consequence of uniparental disomy, as previously shown [2].

**Figure 4:**
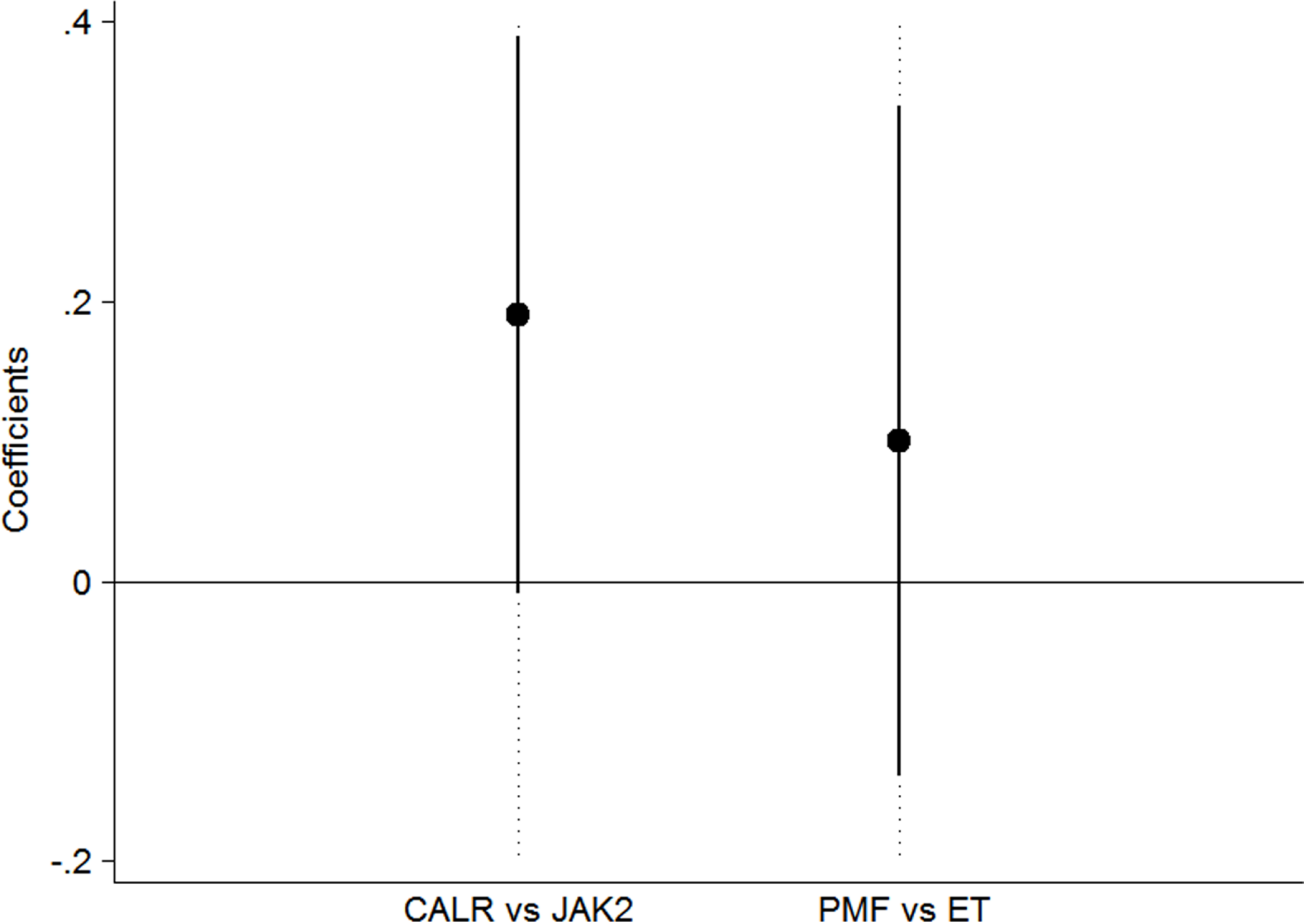
Effect of the mutational status on the slope of mutant burden Multivariate regression with slope as outcome and mutation and diagnosis as covariates. The analysis was carried on the subgroup of patients with essential thrombocythemia (ET) and primary myelofibrosis (PMF) who were untreated to avoid the potential influence of cytoreduction. There was a trend toward a higher increase of mutant burden in *CALR* vs *JAK2* (β = 0.19, *P* = 0.061) with no difference between diagnosis (*P* = 0.419).

Then we repeated the same analysis in a cohort of 226 *JAK2*-mutated patients with similar diagnosis. The median allele burden at last evaluation was not different respect to that at first evaluation, neither in ET (22.9%, range 1%–99.9% vs 23.2%, range 1.7–97.6%, *P* = 0.216) nor in PMF (50.5%, range 1%–99.1% vs 45%, range 1.5–98.6%, *P* = 0.926), although both *JAK2*-mutant ET and *JAK2*-mutant PMF showed a positive slope (0.007 and 0.012 respectively). This might suggest that the clonal expansion in *CALR*-mutant MPN is faster than that observed in *JAK2*-mutant MPN. To test this hypothesis and to evaluate the effect of the type of mutation (*CALR* vs *JAK2*) on the slope of mutant burden adjusting for the effect of diagnosis (ET vs PMF), we performed a multivariate linear regression in all untreated patients (to avoid the potential influence of cytoreduction). Among patients with a positive slope, we observed a trend toward a higher increase of mutant burden in *CALR* vs *JAK2* (β = 0.19, *P* = 0.061) with no difference between diagnosis (*P* = 0.419), as reported in Figure 4.

Taken together, these results suggest that the *CALR*-mutant stem cell would achieve clonal dominance much faster than the *JAK2*-mutant one and confirm the model initially proposed [12] in which the clonal evolution of *CALR*-mutant MPN appears to be mainly associated with a progressive expansion of a mutant heterozygous clone that eventually becomes fully dominant in the bone marrow. Anyway, the sequential evaluation of *CALR*-mutant burden is not recommended in the clinical practice as it lacks clinical utility.

## MATERIALS AND METHODS

Inclusion in the current study required a diagnosis of ET or PMF, availability of clinical data at diagnosis and during follow-up and at least two DNA samples to assess variations of mutant allele burden.

We identified in our database a total of 105 consecutive patients with *CALR*-mutant ET and PMF (76 patients affected with ET, 29 patients affected with PMF). This cohort was compared with a cohort of 226 *JAK2* V617F-mutated consecutive MPN patients with similar diagnosis (183 patients affected with ET, 43 patients affected with PMF). The two cohorts were diagnosed and followed at the Department of Hematology Oncology, Fondazione IRCCS Policlinico San Matteo Pavia from 1985 to 2014. Their clinical characteristics are reported in Table 1. This study was approved by the Ethics Committee of Fondazione IRCCS Policlinico San Matteo, Pavia, Italy. The procedures followed were in accordance with the Helsinki Declaration, and samples were obtained with patients’ written informed consent. Diagnosis of MPN was made in accordance with the criteria in use at the time of the first observation.

**Table 1:** Clinical characteristics of the 105 *CALR*-mutated patients and 226 *JAK2*-mutated patients

	*CALR*-mutated		*JAK2*-mutated	
	ET (*n* = 76)	PMF (*n* = 29)	ET (*n* = 183)	PMF (*n* = 43)
Median age at first evaluation, years (range)	45 (15–77)	46 (18–75)	46 (15–83)	58 (27–82)
Gender, M/F (%)	42/34 (55%/45%)	12/17 (41%/59%)	70/113 (38%/62%)	28/15 (65%/35%)
Median follow-up, years (range)	8.73 (1.07–25.76)	8.16 (0.71–16.67)	9.33 (1.03–28.97)	6.69 (0.84–18.43)
Median interval between first-last sample, months (interquartile range)	45.5 (21.4–91)	39.6 (17.7–78.2)	51.2 (27.3–88.8)	33.0 (17.7–49.0)
Median number of blood samples per patient (range)	2 (2–9)	2 (2–6)	2 (2–11)	2 (2–8)

Granulocytes’ DNA from peripheral blood was used for molecular analysis. *CALR* exon 9 mutations were assessed with Sanger sequencing, as previously reported [4]. The evaluation of *CALR*-mutant burden was done comparing the height of the mutated and wild type peaks. To assess the sensitivity of our technique, genomic DNAs from patients with 50% *CALR* mutant allelic burden (assessed by fragment length analysis [2]) were diluted into DNA from a healthy control. With the use of 40 ng of matrix DNA, dilutions up to 1:8 for type 1 deletion and 1:16 for type 2 insertion still produced detectable and valuable mutant traces, indicating a sensitivity of the method of approximately 6% and 3%, respectively. Sanger data were validated in an independent cohort of 98 *CALR*-mutated patients previously evaluated using fragment analysis (median *CALR*-mutant burden: 47%; range: 10–100) [2]. This cohort included 34 samples of the study cohort. No false-negative samples were found using Sanger analysis and the comparison of *CALR*-mutant burden obtained with the two methods showed a very high Pearson's correlation (ρ 0.94, *P* < 0.001). Sanger sequencing allowed also the genotyping of the rs1049481 [13].

A quantitative real-time polymerase chain reaction (qRT-PCR)-based allelic discrimination assay was employed for the quantification of the *JAK2* V617F allele [14, 15]. The different types of *CALR* mutations were classified as type 1-like and type 2-like according to our previous study [16].

The median interval between first and last sample collection was 45.5 months in ET (interquartile range 21.4–91) and 39.6 months in PMF (interquartile range 17.7–78.2).

Quantitative variables have been summarized in terms of median and range or interquartile range, while categorical variables were described by counts and percentage. The Wilcoxon signed-rank test for paired data was adopted to compare the median value of allele burden at last evaluation respect to the median value at first evaluation. As several patients had more than two sequential evaluations, the variation of allele burden during follow-up was evaluated by the slope of a regression model with allele burden analyzed as outcome and months of follow-up analyzed as independent variable: a slope value higher than 0 refers to an increase of allele burden during time while a negative slope value indicates a decrease of allele burden. The comparison of median slope between two groups was tested by the Mann-Whitney *U* test for unpaired data. The linear regression model was adopted to evaluate the effect of type of mutation and diagnosis on the slope. *P*-values less than 5% were considered significant. Statistical analyses were performed using Stata 12.1 (StataCorp. 2011. Stata Statistical Software: Release 12. College Station, TX: StataCorp LP) software.
